# Hereditary leptomeningeal transthyretin amyloidosis with heterozygous *TTR* mutation: a case report and literature review

**DOI:** 10.1186/s13023-025-03736-x

**Published:** 2025-05-05

**Authors:** Hong-Tao Chen, You-Jun Tian, Jue Zhang, Bing-Rong Xiao, Ke Yang, Ya-Li Zhang

**Affiliations:** Department of Diagnostic Imaging Center, First People’s Hospital of Tianmen, Jingling people Avenue East No. 1, Tianmen City, 431701 Hubei Province China

**Keywords:** Hereditary leptomeningeal transthyretin amyloidosis, Genetic mutation, Transthyretin, Phenotype, Leptomeningeal disease

## Abstract

**Objective:**

This study aimed to characterize the clinical and neuroimaging features of hereditary leptomeningeal transthyretin amyloidosis (hATTR-LA), a dominant inheritance disorder caused by a heterozygous *TTR* gene mutation.

**Methods:**

A comprehensive retrospective evaluation was conducted, incorporating detailed clinical records, multimodal neuroimaging findings, and a systematic review of the literature to contextualize the observations.

**Results:**

The patient was a 55-year-old male who presented with chronic central nervous system symptoms, including sensory-motor peripheral neuropathy and progressive visual impairment. Cerebrospinal fluid analysis revealed elevated protein levels. Neuroimaging showed progressive leptomeningeal hyperdensity on CT and characteristic linear thickening with enhancement of the leptomeninges on MRI, involving both cerebral and spinal regions. Genetic testing confirmed the diagnosis by identifying a heterozygous c.265T > C (p.Y89H) pathogenic variant in exon 3 of the *TTR* gene, classified as pathogenic according to ACMG guidelines.

**Conclusion:**

Multimodal imaging provides valuable, non-invasive insights for diagnosing hATTR-LA, enhancing diagnostic accuracy and informing clinical management of this rare condition.

## Introduction

Hereditary transthyretin amyloidosis (hATTR) represents a rare autosomal dominant genetic disease affecting multiple systems, caused by mutations in the transthyretin (*TTR*) gene [1]. The predominant phenotypes of hATTR include transthyretin amyloid polyneuropathy (hATTR-PN), also known as transthyretin familial amyloid polyneuropathy (TTR-FAP), transthyretin amyloid cardiomyopathy (hATTR-CM), and mixed types. Hereditary leptomeningeal transthyretin amyloidosis (hATTR-LA) is a rare phenotype primarily involving the central nervous system [1–3]. The onset of hATTR-LA typically occurs later in life, and due to the significant variability and lack of specificity in clinical manifestations, tissue pathology, and genetic testing are often conducted in the middle to late stages of the disease, leading to frequent misdiagnoses.

The structural abnormalities and deposition of TTR are caused by mutations in the *TTR* gene. Currently, the *TTR* gene is associated with over 200 phenotypes worldwide, demonstrating phenotype specificity across different genotypes [4]. However, clinical heterogeneity is notably pronounced across various ethnicities and genotypes, as evidenced by variations in prevalence, gender-specific incidence rates, age of onset, and clinical features [5, 6]. This study presents the clinical and imaging data of a patient carrying the pathogenic P.Y89H mutation. Through a comprehensive literature review, the study aims to elucidate the imaging characteristics of hATTR-LA, thereby raising awareness of the disease and contributing to improved prognostic outcomes for affected individuals.

## Materials and methods

### Case presentation

The patient, a 55-year-old male, was admitted to the Department of Neurology at our hospital on May 23, 2023, presenting with “episodic right upper limb numbness persisting for 7 years and intensifying over the past 3 days.” The patient reported an onset of episodic limb numbness and weakness without any apparent cause three days prior, initiating in the right upper limb and progressively extending upwards to the face, accompanied by right-hand finger spasms, reduced mobility, and decreased pain sensation in the right upper limb. One hour later, numbness and weakness in the right lower limb emerged, leading to instability in standing. The patient did not experience dizziness, headache, tinnitus, diplopia, or incontinence.

Clinical data of the patient, including basic information, initial symptoms, laboratory tests, echocardiography, electromyography results, etc., were collected, and blood samples were taken for genetic analysis. This study was conducted in accordance with the Declaration of Helsinki and approved by the Research Ethics Committee of our hospital (ethical batch number: 20240318), and written informed consent was obtained from the participant. All methods were carried out in accordance with relevant guidelines and regulations.

### Past medical history

Seven years prior, the patient experienced sudden numbness in the right upper limb, with the numbness rapidly extending from the fingertips to the right cheek, accompanied by right-hand motor dysfunction, facial asymmetry, and speech difficulties. CT and MRI scans at our hospital revealed no abnormalities, while head and neck CTA indicated atherosclerosis of the right internal carotid artery with slight luminal stenosis. Treatment with aspirin and atorvastatin calcium was initiated (discontinued one-month post-discharge), with no further treatment pursued. Over the subsequent four years, the patient experienced similar episodes 2–3 times annually, with symptoms as described above, usually resolving within 10 min, and never lasting more than 30 min. The gradual onset of tremors in both hands occurred three years ago. Six months prior, the patient experienced two episodes in a single day, presenting with numbness, decreased strength in the left upper limb, and an inability to clench a fist, with full resolution of symptoms within 1–2 h after each episode.

### Physical examination

Upon admission, the systolic blood pressure was 110 mmHg and the diastolic blood pressure was 76 mmHg. The patient was conscious, with pupils equal and round, reactive to light, no cyanosis of the lips, clear breath sounds in the lungs, a heart rate of 72 beats per minute, and no murmurs heard. Cognitive impairment, speech fluency disorder, shallowing of the right nasolabial fold, deviation of the mouth to the left, action tremor in the right upper limb, central tongue position, bilateral hearing loss, diminished visual acuity, and normal limb muscle tone and strength were noted, while no other pathological signs were observed.

### Auxiliary examinations

Lumbar puncture cerebrospinal fluid (CSF) examination: CSF protein 1.47 g/L, CSF cell count, exfoliative cytology showed no abnormalities, complete blood cell count, parathyroid hormone, full set of autoantibodies, 3 items of anticardiolipin antibodies, ANCA spectrum, CBM antibody determination, and full set of rheumatoid tests showed no significant abnormalities.

**Fig. 1:**
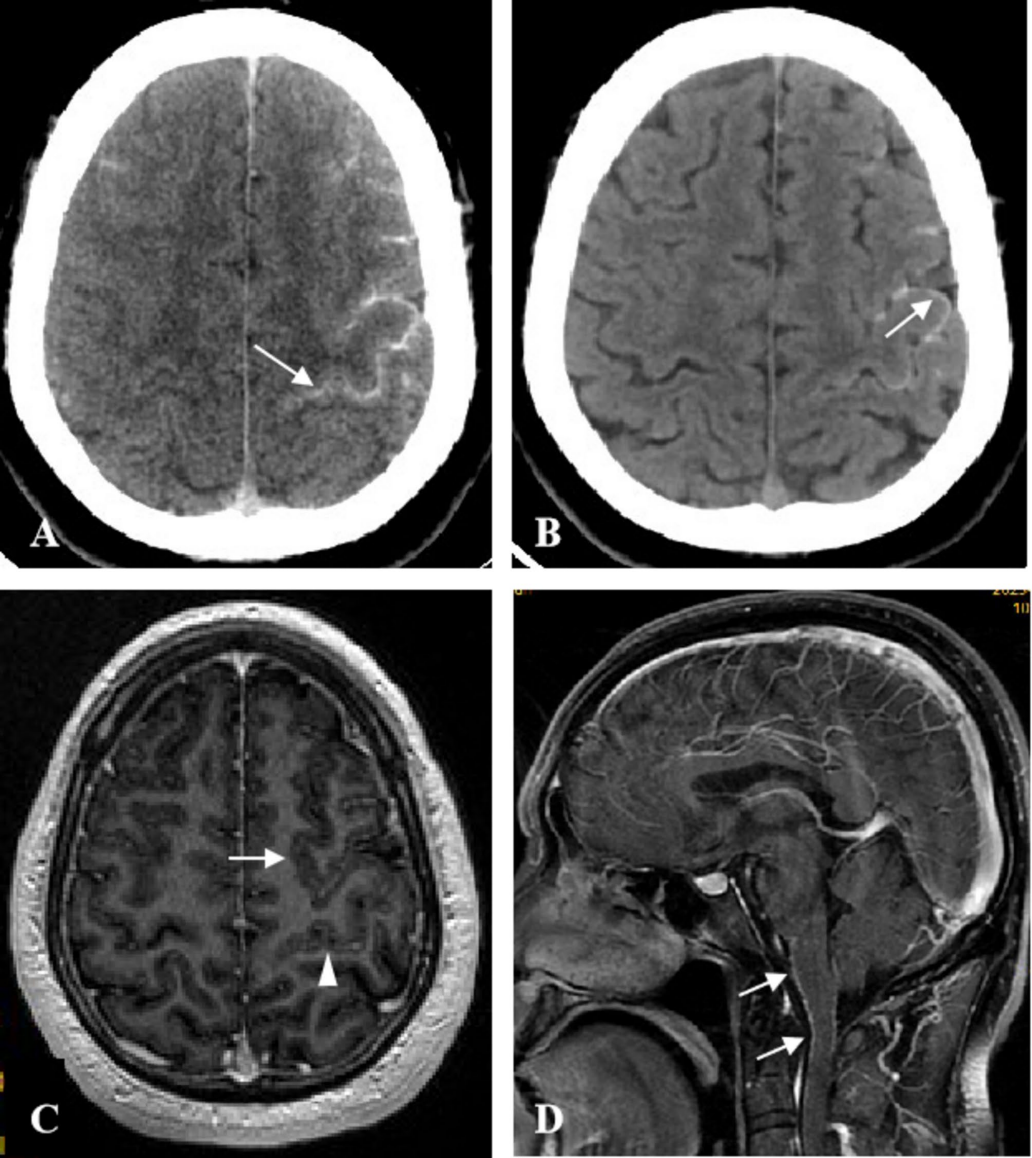
Post-interventional cerebral angiography CT images of the patient showing high density within certain cerebral sulci (**A**, indicated by arrows). Two days later, the high density within the cerebral sulci on CT imaging disappeared, and linear high density appeared in the leptomeninges (**B**, indicated by arrows). The following day, enhanced MRI of the head demonstrated linear enhancement of the leptomeninges and spinal membranes (**C** and **D**, indicated by arrows)

Initial non-contrast head CT (GE Revolution 256-slice; axial acquisition; 0.625 mm slice thickness) demonstrated bilateral hyperdense signals in the lateral and frontal sulci (Fig. 1A). Follow-up CT at 48 h revealed resolution of sulcal hyperdensity with persistent meningeal enhancement (Fig. 1B). Subsequent 3.0T MRI (GE 3T Signa pioneer) was performed using standard sequences: T1/T2-weighted, DWI (b = 1000 s/mm²), SWI, and post-contrast T1 (1.0 mm slice thickness) following gadolinium administration (0.1 mmol/kg). While DWI and SWI sequences showed no significant parenchymal abnormalities, post-contrast T1 imaging demonstrated prominent leptomeningeal enhancement involving the lateral sulci, frontal lobes, brainstem, and upper cervical spinal meninges (Fig. 1C, D). Limb electromyography showed slowed conduction velocity of the right median nerve sensory nerve action potential (SNAP), with no definitive waveform for the left peroneal nerve motor nerve compound muscle action potential (CMAP).

### Genetic analysis

**Fig. 2:**
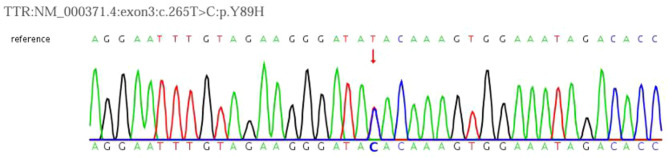
Genetic testing results of the patient. Indication: There is a heterozygous mutation in exon 3 of the TTR gene, c.265T > C (p.Y89H)

Peripheral blood (4 mL) was collected for *TTR* gene Sanger sequencing (PE Applied Biosystems). The analysis identified a heterozygous c.265T > C (p.Y89H) mutation in exon 3 (Fig. 2), which was classified as pathogenic according to ACMG guidelines. The p.Y89H mutation causes a tyrosine-to-histidine substitution, destabilizing transthyretin tetramers and promoting amyloid deposition in the leptomeninges, consistent with the patient’s clinical and imaging findings.

## Discussion

In China, the predominant clinical phenotype of TTR-FAP is characterized by axonal sensory-motor peripheral neuropathy, with a minority of cases involving the meninges [7]. Amyloid deposition in the leptomeninges, arachnoid, and blood vessel walls of the subarachnoid space, observed through meningeal biopsy or autopsy, directly confirms amyloidosis. Peripheral nerve damage in hATTR-LA typically presents mildly or even subclinically, with some patients developing carpal tunnel syndrome or radiculopathy. Approximately one-third of mutations in the TTR gene are associated with vitreous opacities [8]. Laboratory findings indicate elevated CSF protein levels, with normal or slightly elevated cell counts, likely related to amyloid deposition in the leptomeninges and subarachnoid space [9]. Due to the lesser extent of cardiac involvement, this type generally has a better overall prognosis. In this case, the clinical presentation began with central nervous system damage, accompanied by peripheral nerve damage, without evident autonomic, cardiac, or renal involvement, suggesting an advanced stage without renal impairment or characteristic ECG changes, which is consistent with hATTR-LA clinical features.

The gold standard for diagnosing hATTR-LA relies on tissue examination and genetic testing [3, 10, 11]. Over 200 pathogenic *TTR* gene mutations have been identified globally, with the Val30Met mutation being most common among European patients and typically associated with peripheral neuropathy, while in the United States, the Val122Ile mutation is often linked to late-onset cardiomyopathy [11]. In China, the most common genotype is Gly83Arg (19.8%), followed by Val30Met (15.9%) and Val30Ala (7.9%). Of these, 46.03% are classified as the neurological type, 30.16% as the mixed type, and only 2.38% as the cardiac type [6]. Other mutations commonly associated with hATTR-LA include Leu12Pro, Asp18Gly, Ala25Thr, Val30Gly, Ala36Pro, Gly53Glu, Gly53Ala, Phe64Ser, Tyr69His, and Tyr114Cys [12–16]. The p.Y89H mutation, a rare variant not previously reported in China, expands the mutation spectrum of hATTR-LA in this region and enriches the clinical understanding. Research by Yumi Yamada et al. [17] revealed significant levels of *TTR* variants in the CSF of patients with the p.Y89H mutation, which were undetectable in serum, suggesting inefficient secretion of unstable TTR by hepatocytes into the serum, while it is actively secreted into the CSF by choroid plexus cells, likely contributing to leptomeningeal damage. This mutation primarily affects the leptomeninges and eyes, initially presenting with transient focal neurological episodes, epilepsy, vision loss, and later progressing to cognitive impairment and ataxia [18–20].

The natural progression of hATTR-LA follows a distinct imaging trajectory that reflects ongoing amyloid deposition. Initial imaging may appear normal, with leptomeningeal abnormalities developing insidiously over the years [21, 22]. MRI typically detects early changes through meningeal enhancement, which precedes CT-visible calcifications by a significant interval. This pattern was observed in our case, where the enhancement areas exceeded the distribution of calcifications. This temporal sequence suggests MRI’s superior sensitivity in early disease stages [1, 3, 21]. The underlying mechanism involves progressive amyloid disruption of vascular integrity: deposits in leptomeningeal vessels compromise the blood-brain and blood-CSF barriers, permitting contrast leakage that manifests as enhancement [3, 21]. Hirai et al.‘s demonstration of delayed meningeal and CSF enhancement in FAP patients provides strong support for this pathophysiological model [23]. As deposition accumulates, CT eventually detects characteristic calcifications that anatomically correlate with prior MRI enhancement patterns, creating a diagnostic continuum from subtle MRI changes to definitive CT findings. This progression mirrors clinical deterioration, with longer disease duration correlating with more extensive imaging abnormalities [24, 25]. Notably, the slow expansion from focal cerebral to diffuse spinal involvement, as seen in our patient’s serial imaging, reflects the disease’s relentless progression. These observations underscore MRI’s value for early diagnosis in pre-symptomatic stages when therapeutic intervention might be most effective, while CT remains indispensable for confirming advanced disease. The consistent imaging-clinical correlation supports using this temporal progression pattern for monitoring disease course and treatment response.

Due to the pronounced heterogeneity and frequent multisystem involvement of hATTR-LA, early clinical diagnosis presents a significant challenge. However, with ongoing advancements in non-invasive diagnostic techniques, valuable insights for diagnosis can be obtained. The imaging characteristics of hATTR-LA are as follows: (1) Early stages of the disease typically lack distinct imaging features; (2) CT scans reveal leptomeningeal thickening and calcification (rather than in the cerebral sulci), predominantly in the frontal and temporal lobes. Cerebrospinal fluid in the cerebral sulci appears as low-density, best visualized in thin-layer images, with no significant findings in the brain parenchyma; (3) MRI scans generally show no significant findings, but enhancement reveals uniform thickening and smooth linear enhancement of the leptomeninges and spinal membranes, more pronounced on enhanced FLAIR, serving as a key indicator of leptomeningeal involvement; (4) An increased likelihood of contrast agent leakage is observed.

The differential diagnosis of hATTR-LA requires careful consideration of several conditions with overlapping imaging features [26, 27]. (1) First, subarachnoid hemorrhage (SAH) presents acutely with meningeal signs and shows sulcal hyperdensity on CT that typically resolves within days, unlike the persistent leptomeningeal enhancement seen in hATTR-LA. While rare reports suggest hATTR may predispose to SAH, SWI demonstrating hemosiderin deposits and dual-energy CT showing iodine map characteristics help differentiate these entities. (2) Second, cerebral amyloid angiopathy (CAA), though also involving amyloid deposition, primarily affects cortical vessels in elderly patients, presenting with characteristic cortical microbleeds on SWI and cognitive impairment - features absent in our case. (3) Third, infectious etiologies like tuberculous meningitis typically demonstrate basal cistern predilection with thick, nodular enhancement and complications such as hydrocephalus or infarcts, none of which were observed in our patient who also lacked systemic TB symptoms. (4) Fourth, neoplastic meningitis usually shows irregular/nodular enhancement patterns and is typically accompanied by known primary malignancy and positive CSF cytology, all absent in our case. (5) Finally, chronic inflammatory conditions such as neurosarcoidosis generally involve the dura more prominently and are associated with systemic manifestations and elevated inflammatory markers, contrasting with our patient’s isolated neurological presentation. The combination of characteristic imaging findings, including frontotemporal-predominant calcification on CT, smooth linear leptomeningeal enhancement on MRI, and progressive spinal involvement, along with a negative infectious workup, absence of primary malignancy, and positive genetic testing, provides a robust framework for distinguishing hATTR-LA from its mimics. This systematic approach to differential diagnosis is particularly crucial given the condition’s heterogeneous presentation and the importance of early, accurate diagnosis for appropriate management.

This study has several limitations. (1) First, the lack of immediate post-angiography spectral imaging due to equipment constraints prevented direct visualization of contrast extravasation, leaving open the question of whether early-stage spectral imaging could detect initial amyloid deposition. (2) Second, the single-case design limited our ability to characterize early MRI enhancement patterns, and the absence of delayed FLAIR sequences precluded assessment of meningeal enhancement evolution and CSF enhancement dynamics. (3) Additionally, the observed spinal MRI enhancement lacked corresponding CT calcifications, necessitating longer follow-up to determine whether this represents early disease or a distinct pattern. The absence of longitudinal imaging also restricted our ability to track amyloid progression over time, highlighting the need for prospective studies with serial imaging. (4) Finally, while literature reports associate the p.Y89H mutation with contrast-induced neurological complications [16, 18, 21], our study could not establish whether this risk is mutation-specific, a question warranting larger cohort studies.

In recent years, clinical guidelines have increasingly recognized the role of ethnic and geographic variations in hATTR amyloidosis, leading to the adoption of more tailored diagnostic strategies [28–30]. Significant advancements in treatment have been made, particularly with CRISPR-based TTR silencing therapies like NTLA-2001, which has demonstrated promising results in clinical trials by significantly reducing serum TTR levels through targeted gene editing while maintaining a favorable safety profile [31]. Additionally, AI-powered multimodal imaging has emerged as a powerful diagnostic tool, enhancing the accuracy of early detection. Collectively, these innovations, ranging from improved diagnostic approaches to cutting-edge treatments, are significantly advancing the early identification and management of hATTR, including its leptomeningeal variant (hATTR-LA).

## Conclusion

In conclusion, hATTR-LA is an autosomal dominant hereditary disease, and its diagnosis relies on typical clinical presentations, family history, pathology, genetic testing, and multimodal imaging. This non-invasive approach offers valuable insights into diagnosing hATTR-LA. Furthermore, the clinical heterogeneity of *TTR* mutations varies significantly across different ethnicities and genotypes. Considering these ethnic and geographical disparities, advocating for genetic screening in patients with unexplained leptomeningeal thickening is essential to enhance early detection and improve management strategies for this condition.

## Data Availability

Data will be made available on request.
